# Barriers and enablers to implementing and using clinical decision support systems for chronic diseases: a qualitative systematic review and meta-aggregation

**DOI:** 10.1186/s43058-022-00326-x

**Published:** 2022-07-28

**Authors:** Winnie Chen, Claire Maree O’Bryan, Gillian Gorham, Kirsten Howard, Bhavya Balasubramanya, Patrick Coffey, Asanga Abeyaratne, Alan Cass

**Affiliations:** 1 Menzies School of Health Research, Charles Darwin University, PO Box 41096, Casuarina, NT 0811 Australia; 2 School of Public Health, Faculty of Medicine and Health, University of Sydney, Sydney, NSW Australia

**Keywords:** CDS, CDSS, Clinical decision support systems, Chronic disease, Evaluation, Health system, Implementation, Meta-aggregation, Qualitative, Systematic review

## Abstract

**Background:**

Clinical decision support (CDS) is increasingly used to facilitate chronic disease care. Despite increased availability of electronic health records and the ongoing development of new CDS technologies, uptake of CDS into routine clinical settings is inconsistent. This qualitative systematic review seeks to synthesise healthcare provider experiences of CDS—exploring the barriers and enablers to implementing, using, evaluating, and sustaining chronic disease CDS systems.

**Methods:**

A search was conducted in Medline, CINAHL, APA PsychInfo, EconLit, and Web of Science from 2011 to 2021. Primary research studies incorporating qualitative findings were included if they targeted healthcare providers and studied a relevant chronic disease CDS intervention. Relevant CDS interventions were electronic health record-based and addressed one or more of the following chronic diseases: cardiovascular disease, diabetes, chronic kidney disease, hypertension, and hypercholesterolaemia. Qualitative findings were synthesised using a meta-aggregative approach.

**Results:**

Thirty-three primary research articles were included in this qualitative systematic review. Meta-aggregation of qualitative data revealed 177 findings and 29 categories, which were aggregated into 8 synthesised findings. The synthesised findings related to clinical context, user, external context, and technical factors affecting CDS uptake. Key barriers to uptake included CDS systems that were simplistic, had limited clinical applicability in multimorbidity, and integrated poorly into existing workflows. Enablers to successful CDS interventions included perceived usefulness in providing relevant clinical knowledge and structured chronic disease care; user confidence gained through training and post training follow-up; external contexts comprised of strong clinical champions, allocated personnel, and technical support; and CDS technical features that are both highly functional, and attractive.

**Conclusion:**

This systematic review explored healthcare provider experiences, focussing on barriers and enablers to CDS use for chronic diseases. The results provide an evidence-base for designing, implementing, and sustaining future CDS systems. Based on the findings from this review, we highlight actionable steps for practice and future research.

**Trial registration:**

PROSPERO CRD42020203716

**Supplementary Information:**

The online version contains supplementary material available at 10.1186/s43058-022-00326-x.

Contributions to the literature

- Clinical decision support interventions are increasingly used to care for people with chronic diseases. We sought to understand the reasons why some projects are successfully implemented, whereas others fail to gain user uptake or acceptance in clinical settings.
- We summarise existing primary research examining the success and failure factors of clinical decision support implementations. We found that factors affecting clinical decision support uptake related to clinical context, user, external context, and technological factors.
- Our summary of recommendations is useful in guiding health practitioners and policy makers in designing, implementing, and sustaining clinical decision support systems.

## Background

### Introduction

Clinical decision support (CDS) refers to a wide range of tools that present clinical or patient-related information, “intelligently filtered or presented at appropriate times” to enhance healthcare processes and patient outcomes [1]. Modern computerised CDS systems are data-driven and utilise individual patient data from existing electronic health records (EHRs) [2]. EHR data is extracted and processed via algorithms, and decision support outputs are displayed to users within EHRs themselves or in standalone web-based applications. Provider-facing CDS functions include tools for clinical documentation, data presentation, order or prescription creation, protocol or pathway support, reference guidance, and alerts or reminder [3, 4]—typically, CDS systems incorporate one or more of these functions.

As early as 1998, decision support was identified as a fundamental area of high-quality chronic disease management within the Chronic Care Model [5]. The current COVID-19 pandemic has accelerated the need for virtual models of care in addition to traditional face-to-face chronic disease care [6]; improving chronic disease care through technology-enabled care models is also a goal of the current Australian national digital health strategy [7]. EHR-based CDS is a digital health intervention that can facilitate the whole spectrum of chronic disease care from screening and diagnosis; to management and optimisation of care pathways; to ongoing individual and population-level disease monitoring.

CDS has the potential to improve health process outcomes, such as adherence to recommended preventative care measures [8–12]. However, meta-analyses of CDS intervention studies show substantial heterogeneity in effect sizes [8–10]. Furthermore, widespread adoption of CDS systems has not been achieved [13], and access to CDS does not guarantee user uptake or acceptance in clinical settings [14–16]. For example, a review of 23 studies examining medication CDS systems found that between 46 and 96% of medication alerts were overridden by clinicians [15]. Other studies have found that an overload of CDS alerts can even contribute to burnout amongst time-poor clinicians [17]. Why do some CDS implementations succeed, whereas others fall short of expectations [18]? Inconsistent CDS uptake stems from technical issues during development, and contextual challenges to implementing the technology [19, 20]. From a technical perspective, CDS development requires complex clinical logic to be converted into computer-executable algorithms, necessitating skills in both medicine and informatics [13]. From an implementation perspective, CDS interventions commonly encounter user and organisational barriers.

Despite a sizeable volume of perspective pieces on why CDS undertakings succeed or fail, there remains few contemporary qualitative systematic reviews on the topic. Several systematic reviews have investigated CDS uptake using a quantitative approach, reporting user satisfaction scores, or reporting meta-regression results to identify factors statistically associated with positive CDS outcomes [8, 9, 21, 22]. Other reviews investigate CDS uptake by summarising the experience of healthcare providers—through narrative or thematic meta-synthesis of primary qualitative research findings [18, 23–28]. Two of these qualitative systematic reviews summarised early primary research studies in the field from the 1990s and 2000s [18, 25]. More recent reviews have been conducted to investigate barriers and enablers to CDS uptake, but have predominantly focussed on a single type of CDS—for example, CDS that is medication-related [24, 25], or CDS systems that are alert-based [26]. One 2015 study by Miller et al uses a comprehensive definition of computerised CDS—however, the authors only selected 9 of 56 eligible qualitative studies for inclusion in the final synthesis, as studies of limited methodological quality were excluded from the qualitative synthesis [23].

Building on previous studies, we sought to conduct a systematic review of barriers and enablers affecting EHR-based CDS adoption for chronic disease care. Barriers and enablers are determinants of practice that prevent or enable knowledge translation [29, 30]. A barrier adequately addressed becomes a facilitator for health intervention implementation. A synthesis of barriers and enablers across multiple studies can bring valuable perspectives not found within an individual study. A myriad of qualitative synthesis methods have been developed, each with its own epistemological stance and stated purpose [31, 32]. Broadly speaking, qualitative synthesis methods lie on a continuum between primarily aggregative to primarily interpretive approaches [31, 33]. Aggregative approaches such as meta-aggregation seeks to pool findings across studies, mirroring the meta-analysis process used in quantitative studies. In contrast, interpretative approaches such as meta-ethnography are grounded in social science research traditions and seek to re-interpret primary research findings to develop theories and frameworks [34, 35]. We use a JBI (formerly Joanna Briggs Institute) meta-aggregation approach for synthesising qualitative evidence for two main reasons—firstly, aggregative rather than interpretive synthesis methods is most appropriate for qualitative data in the CDS field, with its limited availability of contextually “thick” and conceptually “rich” qualitative findings [36, 37]; secondly, meta-aggregation best aligns with our review objective of identifying actionable steps at a practice level. A preliminary search of PROSPERO, Cochrane Database of Systematic Reviews, JBI database and Medline did not reveal published or studies in progress that conducted a similar scope of work.

### Objectives

This qualitative systematic review aims to describe healthcare provider experiences of implementing, using, evaluating, and sustaining EHR-based CDS interventions for chronic disease care. Barriers and enablers of providers will be described from the perspectives of individual clinicians, and healthcare services.

## Methods

The systematic review is registered on PROSPERO (CRD42020203716). The overall approach to this CDS review was a mixed method review incorporating qualitative, effectiveness, and economic evaluation components. Only the qualitative component of the systematic review is reported in this paper, and the effectiveness and economic reviews will be reported separately. Methods for the qualitative systematic review are informed by JBI methodology for systematic reviews of qualitative evidence, and JBI methodology for text and opinion [38]. The review was reported according to Preferred Reporting Items for Systematic reviews and Meta-Analyses (PRISMA) guidelines—see Additional file 1.

### Searches

Databases searches included PubMed (Medline), EBSCOHOST (CINAHL, APA PsychInfo, EconLit), and Web of Science. MeSH terms and title/abstract search terms included synonyms of “clinical decision support systems,” “cardiovascular disease,” “diabetes,” “chronic kidney disease,” “hypertension,” and “hypercholesterolaemia.” The full search strategy for PubMed (Medline) is outlined in Additional file 2. Studies were restricted to English language studies from January 2011 to January 2021. Studies prior to 2011 have been adequate covered by previous reviews [8, 18, 25] and our review targeted contemporary EHR-based CDS system implementations—hence, we restricted the search to studies published within the past decade.

### Study inclusion and exclusion criteria

#### Population

Our population of interest was individual clinician (e.g. doctors, nurses, pharmacists) or other health service staff (e.g. clinic managers) using EHR CDS systems as a point-of-care tool for patients with one or more of five chronic diseases. Even though CDS can be both provider-facing and patient-facing, we focussed on provider perspectives since providers are the main implementers and users of CDS systems. The five related chronic diseases were chronic kidney disease, cardiovascular disease, diabetes, hypertension, and hypercholesterolaemia. CDS targeting the whole spectrum of chronic disease care was considered—including but not limited to CDS used for screening and diagnosis, pathway support, pharmacological management, and non-pharmacological management of chronic diseases. Basic EHR-based CDS functions with limited scope—such as single medication CDS (e.g. warfarin dosing) and simple recall alerts—were excluded from this study.

#### Phenomena of interest and context

The phenomena of interest were healthcare provider experiences of chronic disease CDS systems, focussing on barriers and enablers to implementing, using, evaluating, and sustaining CDS interventions. We sought to understand “real-world” provider experiences and therefore, CDS prototypes not used in clinical settings were excluded from the study. The contexts of interest were non-acute settings delivering chronic disease care, which included primary care, specialist outpatient, and community healthcare services. CDS systems used in emergency or other acute settings only, such as CDS for inpatient management of blood glucose levels, were excluded from this study; this is because optimal healthcare processes to address chronic diseases are inherently different from that of acute hospital services [5, 39]. See Additional file 3 for a detailed inclusion and exclusion criteria.

#### Other eligibility criteria

Primary research studies with CDS systems implemented for clinical use were included—thus, CDS research protocols and articles describing methods of CDS development without real-world implementation were excluded. Secondary research such as perspective pieces and systematic reviews were excluded. Study designs considered included qualitative studies and other evaluation studies with a qualitative inquiry component—these included studies conducting interviews, surveys, focus group discussions, and mixed methods studies. For the purposes of this review, “qualitative studies” referred to studies with an explicit qualitative methodological approach (e.g. phenomenology). “Other evaluation studies” refers to studies incorporating a qualitative inquiry component (e.g. user feedback study) but without an explicit qualitative methodological approach. We include both types of studies in our review, recognising that evidence from both formal qualitative studies and “other evaluation studies” with qualitative findings can enrich a synthesis [37].

### Study selection

Identified citations were uploaded into Covidence software (Veritas Health Innovation, Melbourne, Victoria, Australia) [40] for screening and article selection. Title and abstracts were screened independently by two independent reviewers (WC, and BB or PC). Conflicts were resolved by reaching a consensus between the two reviewers, and where this was not possible, conflicts were resolved by a third team member. Full text screening was conducted, and studies were classified into qualitative, effectiveness, or economic categories by one reviewer (WC). Reasons for exclusion at full text stage were recorded. Citations for inclusion were imported into the JBI System for the Unified Management, Assessment and Review of Information (JBI SUMARI) software (JBI, Adelaide, Australia) [41] for critical appraisal and data extraction.

### Study quality assessment

Critical appraisal of methodology quality was conducted using the JBI Critical Appraisal Checklist for Qualitative Research 2017 [38] by two reviewers (WC and CO). A random 25% sample of included studies had methodology quality assessed by both reviewers independently and disagreements were resolved through consensus. This review took an inclusive approach to incorporate a broad range of healthcare provider experiences—therefore, study quality assessment was conducted but studies were not excluded from data extraction or synthesis based on methodological quality scoring.

### Data extraction and synthesis

**Fig. 1:**
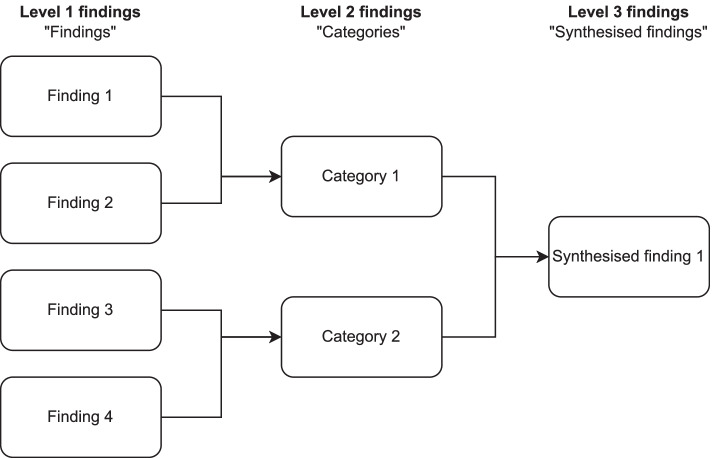
Meta-aggregation and hierarchy of findings

We based our qualitative data extraction and synthesis on the JBI meta-aggregation approach [38]. Meta-aggregation is philosophically grounded in pragmatism and transcendental phenomenology [31]. Pragmatism roots are reflected in an emphasis to identify “lines of action” for policy and practice, which contrasts with other synthesis methods (e.g. meta-ethnography) that have a focus on mid-level theory generation [42]. The influence of transcendental phenomenology is seen in meta-aggregation bracketing, where findings are extracted as given without re-analysis or re-interpretation based on prior concepts regarding the phenomena of interest [43]—through bracketing, meta-aggregation seeks to aggregate primary study findings in a way that represents the original authors’ intended means, with minimal influence from the reviewer [43, 44]. The qualitative meta-aggregation process generates a hierarchy of findings, categories, and synthesised statements—pictorially represented in Fig. 1. In meta-aggregation, level 1 findings are extracted findings (e.g. themes) reported verbatim from primary research authors, which are backed with supportive illustrations (e.g. interview excerpts). Level 2 findings are reviewer-defined categories, which group two or more level 1 findings based on similarities in meaning. Level 3 findings are synthesised statements, which represent the collective meaning of categories and provide recommendations for practice [38, 44].

For data extraction, two reviewers extracted study characteristics and qualitative findings from included studies using JBI SUMARI software (WC and CO) [41], according to a modified JBI meta-aggregation approach [38]. Study characteristics were extracted using the standardised JBI SUMARI tool and included method of data collection and analysis, study setting, phenomena of interest, and participant characteristics. Findings referred to verbatim extracts of the author’s interpretation of qualitative or mixed methods results; illustrations were direct quotations or other supporting data from the primary study (e.g. interviews, surveys). In addition to primary qualitative research findings, verbatim author text and opinion describing primary quantitative research data (e.g. survey data) were also extracted for synthesis. A level of credibility from unequivocal (U), credible (C) to not supported (N) was assigned to each finding. Qualitative findings were pooled—only unequivocal (U) and credible (C) findings were grouped into categories, which were meta-aggregated into synthesised findings. Not supported (N) findings are presented separately.

## Results

### Included studies

**Fig. 2:**
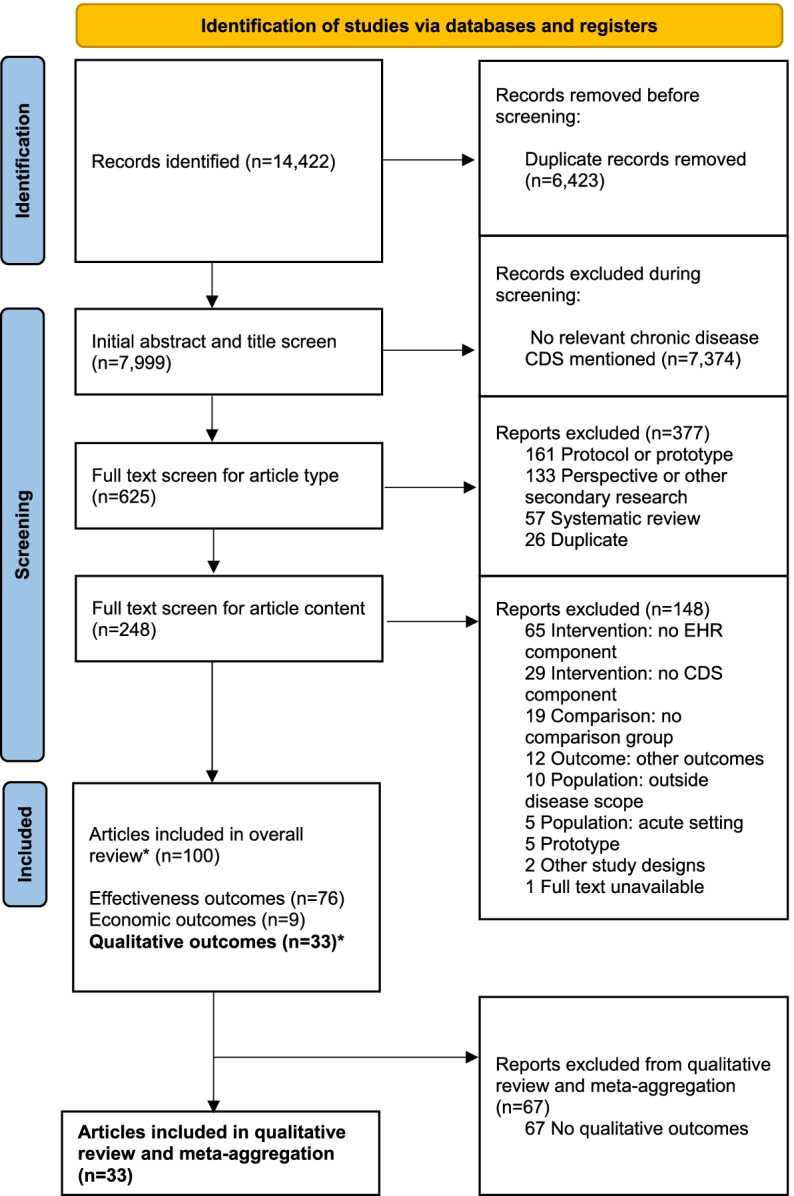
PRISMA flow diagram. Abbreviations: CDS—Clinical decision support; EHR—Electronic health record. *Articles may include one or more outcomes. Bold: Only articles with qualitative outcomes were included in this manuscript, studies with effectiveness or economic outcomes are reported separately

Abstract and title screening was conducted for 7999 citations. Articles were excluded at this stage (*n* = 7374) mainly due to an absence of a chronic disease CDS mentioned in the abstract and title. Full text review was conducted for 625 articles. Reasons for exclusion were as follows: 377 based on article type (e.g. protocol, non-primary research articles) and 148 based on article content. The most common reasons for exclusion based on article content were that the CDS did not have an EHR component (*n* = 65) or that the EHR did not have a CDS component (*n* = 29). Thirty-three studies met the inclusion criteria for the qualitative outcome component of this systematic review, of which 13 were qualitative studies and 20 were other evaluation studies with qualitative findings. All 33 studies were included for data extraction and qualitative evidence synthesis (meta-aggregation). See also Fig. 2 for PRISMA flow diagram [45]. Effectiveness and economic evaluation components of the systematic review will be reported separately.

### Study quality assessment

Overall methodological quality was moderate for the 13 qualitative studies—main issues identified during quality assessment related to limited descriptions of methodological approach (JBI Checklist for Qualitative Research Q1 and Q2) and limited description of researcher context (Q6 and Q7). For the 20 other evaluation studies with qualitative findings, Q1 to Q7 were considered not applicable. The main issue identified in other evaluation studies was that few (*n* = 6) had evidence of ethics approval. See Additional file 4 for methodological quality of included studies.

### Characteristics of included studies

**Fig. 3:**
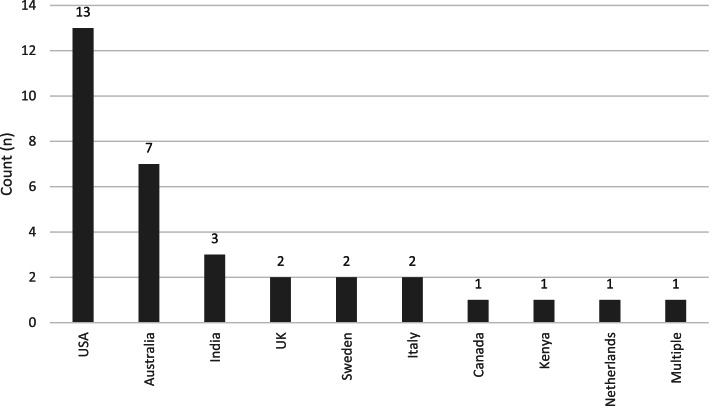
Country of study

**Fig. 4:**
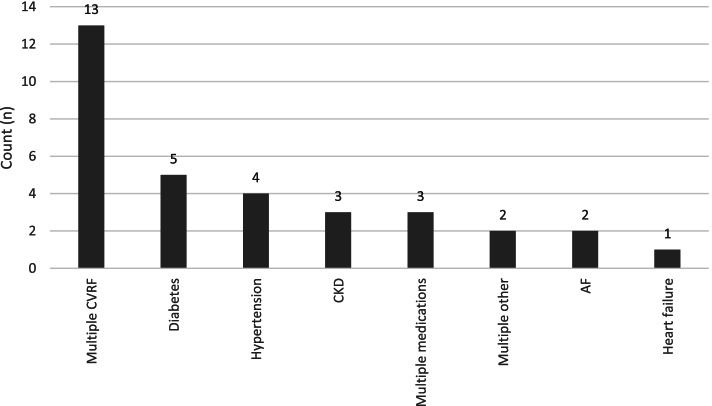
CDS disease focus. Abbreviations: CVRF—cardiovascular risk factors; CKD—chronic kidney disease; AF—atrial fibrillation

The highest number of studies were conducted in USA (*n* = 13), Australia (*n* = 7), and India (*n* = 3) (Fig. 3). Year of publication ranged from 2011 to 2020, with the highest number of articles (48%) published in 2018 and 2019. The most common disease focus of the CDS systems were cardiovascular risk factors (*n* = 13), followed by diabetes (*n* = 5), hypertension (*n* = 4), and multiple medications (*n* = 4) (Fig. 4). The majority of studies were conducted in primary care (*n* = 27)—which referred to general practices, community health clinics, and primary care practices. The remaining studies were conducted in specialist outpatients (*n* = 3) and multiple settings (*n* = 3). A range of CDS types were included: from screening and diagnosis, to pathway support, pharmacological management, and non-pharmacological management. See Table 1 for characteristics of included studies.

**Table 1:** Characteristics of included studies

Study	Study type, method for data collection and analysis	Country	Phenomena of interest	Setting/ context/ culture	Participant characteristics and sample size
Abimbola et al. 2019 [46]	Qualitative study Data collection—mixed methods: primary data from ex post interviews, secondary data from existing surveys and interviews Data analysis—deductive coding using “Nonadoption, Abandonment, Scale-up, Spread, and Sustainability (NASSS) framework” [47]	Australia	CDS tool for treatment of cardiovascular risk: factors influencing uptake and sustained use	Primary care—general practice	Primary data—interview of 5 members of the programme evaluation team (3 chief investigators, 1 project manager, 1 PhD student) Secondary data—sample size not stated, comprised of a range of participating GPs and health professionals across several previous qualitative studies
Ballard et al. 2017 [48]	Other evaluation Data collection—surveys	USA	CDS tool for diabetes medications and statins: use of tool and barriers to use amongst providers	Primary care—1 clinic	262 comprised of 42 nurse practitioners, 8 physician assistants, 120 physicians in training, 92 physicians
Chiang et al. 2017 [49]	Other evaluation Data collection—interviews	Australia	CDS tool for cardiovascular risk evaluation and management: acceptability and feasibility of the tool	Primary care—general practice—1 clinic	5 GPs over 1 day
Cho et al. 2014 [50]	Other evaluation Data collection—user usage data	USA	CDS tool with medication alerts and drug suggestions for patients with renal insufficiency: appropriateness of overriding alerts	Primary care—general practice—36 clinics	584 prescribers over 3 years
Conway et al. 2018 [51]	Other evaluation Data collection—surveys, focus groups	UK	CDS tool for diabetes management and prescribing: use of tool, barriers to use and feasibility in practice	Primary care, specialist outpatients—number of clinics not stated	105 health care professionals (GPs/nurses) over 3 months
Dagliati et al. 2018 [52]	Other evaluation Data collection—surveys, focus groups	Italy	CDS tool for cardiovascular risk calculation: usability and impact on clinical activity	Primary care, specialist outpatients—number of clinics not stated	6 doctors, 1 health care manager
Dixon et al. 2016 [53]	Other evaluation Data collection—surveys	USA	CDS tool for diabetes and cardiovascular addressing risk factors and medication management: use and perception of tool	Primary care—community health centres—3 clinics	6 healthcare providers after using CDS for 9 months
Fico et al. 2019 [54]	Qualitative study Data collection—surveys, focus groups Data analysis—mixed methods evaluation using the “Center for eHealth Research and Disease Management (CeHRes) Roadmap” framework [55]	Italy	CDS tool for diabetes management: user needs, requirements and organisational conditions for successful design and adoption of tool	Specialist outpatients—1 endocrinology clinic	90 healthcare professionals after 2 weeks of CDS use
Gill et al. 2019 [56]	Other evaluation Data collection—surveys, interviews	USA	CDS tool for diabetes management: facilitators and barriers to implementing tool and achieving optimal management	Primary care—12 clinics	10 staff (physician and clinic staff members) after 1-year follow-up period of CDS
Gold et al. 2019 [57]	Qualitative study Data collection—interviews (in-person and phone) Data analysis—inductive approach to thematic analysis, findings presented with “Consolidated Framework for Implementation Research (CFIR)” [58]	USA	CDS tool for ACE inhibitor/ARB and/or statin prescribing (with 3-tiered implementation support): factors influencing effectiveness of tool in improving prescribing practices	Primary care—29 clinics	Number of providers interviewed not stated, interviews from 16 to 33 months of the study
Helldén et al. 2015 [59]	Other evaluation Data collection—surveys, focus group	Sweden	CDS medication tool for renal drug dosing: ease of use and perceived usefulness of tool	Primary care—general practice—2 clinics	8 GPs using CDS for up to 13 months
Holt et al. 2018 [60]	Qualitative study Data collection—interviews (in person or by phone) Data analysis—inductive approach to thematic analysis	UK	CDS for anticoagulation in atrial fibrillation: acceptability and usability of the tool	Primary care—general practice—23 clinics	7 GPs and 15 patients following 6 months use of CDS
Jindal et al. 2018 [61]	Other evaluation Data collection—interviews	India	CDS tool for hypertension and diabetes management: barriers to implementation and use, and solutions to these challenges	Primary care—community health centres—5 clinics	5 physicians and 5 nurses following 2-month pilot
Kumar et al. 2018 [62]	Qualitative study Data collection—interviews Data analysis—inductive approach to thematic analysis	Australia	CDS tool for diabetes management: usability of tool and general views of GPs	Primary care—general practice—4 clinics	6 GPs after using CDS tool for 2 weeks
Litvin et al. 2016 [63]	Other evaluation Data collection—group interviews	USA	CDS tool for chronic kidney disease screening and management: facilitators and barriers to use	Primary care—12 clinics	25 physicians following 12 months of using CDS
Lopez et al. 2019 [64]	Other evaluation Data collection—interviews	USA	CDS tool for hypertension management: acceptability and feasibility of intervention	Primary care—13 clinics	Number of providers interviewed not stated—interviewed following 12 months of CDS use
Lugtenberg et al. 2015 [65]	Other evaluation Data collection—surveys, focus groups	Netherlands	CDS tool for heart failure management: attitudes and perceived barriers	Primary care—general practice—231 clinics	24 primary care practitioners (for focus group), 112 GPs and 52 nurses (for survey) following 12 months of CDS use
Majka et al. 2019 [66]	Other evaluation Data collection—surveys	USA	CDS tool for cardiovascular risk and statin prescribing in rheumatoid arthritis patients: attitudes and practices towards the tool	Specialist outpatients—1 rheumatology clinic	12 clinicians (including rheumatologists, rheumatology fellows, advanced practice nurses, physician assistants) after CDS use for 14 months
Meador et al. 2018 [67]	Other evaluation Data collection—surveys, telephone interviews	USA	CDS tool for hypertension diagnosis: perceptions of successes, challenges, and future needs	Primary care—10 clinics	9 project leads following 17 months of CDS tool use
Millery et al. 2011 [68]	Qualitative study Data collection—interviews Data analysis—inductive approach to thematic analysis	USA	CDS tool for hypertension detection and management: satisfaction, perceived usefulness of tool and facilitators of change	Primary care—4 clinics	16 providers 3-4 months after using CDS tool, 6 key informants (leadership positions and staff) 5-6 months after CDS tool implemented
O’Reilly et al. 2014 [69]	Other evaluation Data collection—surveys	Canada	CDS tool for diabetes management: usability and satisfaction	Primary care—family practice—9 clinics	21 participants pre, and 9 participants post 12 months CDS implementation
Orchard et al. 2019 [70]	Qualitative study Data collection—interviews Data analysis—mixed methods based on realist evaluation framework [71], inductive approach to thematic analysis	Australia	CDS tool for atrial fibrillation screening: circumstances in which the programme worked or not	Primary care—general practice—16 clinics	21 GPs, 13 nurses, 11 practice managers following approximately 40 months post CDS implementation
Patel et al. 2018 [72]	Qualitative study Data collection—surveys, interviews Data analysis—mixed methods with inductive thematic analysis and interpretation based on “normalisation process theory (NPT)” framework [73]	Australia	CDS tool for cardiovascular risk screening and management: impact and factors affecting impact	Primary care—4 general practices and 2 Aboriginal community controlled health services	19 total: 9 GPs, 4 practice managers, 3 Aboriginal health workers, 1 practice nurse, 1 health information office, and 1 administrative assistant/practice manager following 17 months post CDS implementation
Peiris et al. 2011 [74]	Qualitative study Data collection—interviews (in person or by phone) and survey Data analysis—inductive approach to thematic analysis	Australia	CDS tool for CVS risk management: attitudes towards tools and impact on consultation	Primary care—general practice—8 clinics	21 GPs following pilot of CDS
Praveen et al. 2014 [75]	Qualitative study Data collection—interviews, focus group discussions and surveys Data analysis—inductive approach to thematic analysis	India	CDS tool for cardiovascular risk management: barriers and enablers to use of tool	Primary care—11 villages (field tested) and 3 primary health care centres	11 non-physician health care workers and 3 primary care physicians following 1 month of CDS use
Raghu et al. 2015 [76]	Other evaluation Data collection—surveys	India	CDS tool for cardiovascular risk: usability of tool	Primary care—field visits in 3 villages	3 GPs and 11 healthcare workers using the CDS tool for 1 month
Regan, 2017 [77]	Other evaluation Data collection—surveys	USA	CDS tool for chronic kidney disease detection, evaluation, and referral: knowledge and attitudes towards tool	Primary care—11 clinics	55 physicians, 17 nurse practitioners, and 8 physician assistants after using CDS for 3 months
Romero-Brufau et al. 2020 [78]	Other evaluation Data collection—surveys	USA	CDS for glycaemic control in diabetes: barriers and facilitators of using tool	Primary care—3 clinics	Physicians, registered nurses, licensed practical nurses, social workers (number not specified) after using CDS tool for 3 months
Shemeikka et al. 2015 [79]	Other evaluation Data collection—surveys, focus groups	Sweden	CDS tool for prescribing in patients with reduced renal function: usefulness and users’ needs	Primary care, specialist outpatients, and inpatient unit—1 geriatric clinic, 1 internal medicine ward, 2 outpatient healthcare centres	38 physicians after CDS use for 5 weeks
Singh et al. 2018 [80]	Qualitative study Data collection—interviews (in person or by phone) Data analysis—deductive coding using “Rogers’ diffusion of innovation” theory [81]	India and Pakistan	CDS tool for diabetes: provider perceived benefits, challenges, and value of tool	Specialist outpatients—10 diabetes clinics	39 interviews with physicians (endocrinologists) including 19 pre-implementation, 9 1-year interim, and 11 end-of study interviews at 36 months
Sperl-Hillen et al. 2018 [82]	Other evaluation Data collection—surveys	USA	CDS too for cardiovascular risk: use, provider satisfaction and perception	Primary care—20 clinics	102 primary care practitioners before and 18 months after using CDS
Vedanthan et al. 2015 [83]	Qualitative study Data collection—focus group, interviews Data analysis—mixed methods with both inductive approach to thematic analysis and deductive coding (no theoretical framework stated)	Kenya	CDS tool for hypertension: feasibility, barriers	Primary care—rural clinics (number of clinics not specified)	12 nurses following 1 month use of CDS
Wan et al. 2012 [84]	Qualitative study Data collection—surveys, phone interviews Data analysis—inductive approach to thematic analysis	Australia	CDS tool for diabetes management: use, impact, and barriers to use amongst providers	Primary care—general practice (number of clinics not specified)	22 GPs and 2 practice nurses using the CDS for at least 6 weeks

The median number of provider participants was 19 (IQR 10 to 63); 4 studies did not state the number of participants included. Methods used for data collection included interviews (*n* = 11), surveys (*n* = 7), or more than one method (*n* = 14). Those using more than one data collection method mainly used surveys and interviews, or surveys and focus groups. For data analysis, 6 studies used an inductive approach to thematic analysis and 2 used deductive coding—utilising the NASSS framework [46] and Rogers’ diffusion of innovation [80]. The remaining qualitative studies used other evaluation approaches, drawing on theoretical frameworks such as the Consolidated Framework for Implementation Research (CFIR) [57], realist evaluation framework [70], Normalisation Process Theory (NPT) [85], and Centre for eHealth Research (CeHRes) Roadmap [54].

### Review findings and meta-aggregation

From included articles, 177 findings were extracted on barriers and enablers to implementation and use of CDS. Out of these findings, 40 (22%) were unequivocal, 65 (37%) were credible, and 72 (41%) were not supported. For the full list of findings with illustrations, see Additional file 5. The findings were grouped into 29 categories, which were synthesised into 8 findings. Findings, categories, and synthesised findings are presented in Tables 2, 3, 4, and 5.

**Table 2:** Clinical barriers and enablers

	Finding	Category	Synthesised finding
**Clinical context barriers**	Interfered with communication (*n* = 3)	Interference with communication, priorities, and clinical relationship during the consult	Providers experienced clinical context barriers with the interference to clinician-patient communication, lack of CDS applicability, particularly with regard to inappropriate application of guidelines
	Distorted priorities (*n* = 2)		
	Patient’s own agenda		
	Lack of applicability	Lack of applicability due to limited number of conditions addressed or patient factors	
	Information not included		
	[Limited] number of conditions		
	Health literacy [of patient]		
	Cookbook medicine	Guidelines applied indiscriminately to patients	
	Blanket recommendations		
	Conflicting guidelines		
**Clinical context enablers**	Systematic consistent care (*n* = 3)	Support systematic and structured processes, improving quality of care	Providers experienced clinical context enablers where CDS supported structured quality care, facilitated discussions with patients, improved clinical judgment, and presented useful clinical knowledge
	Improved quality of care (*n* = 2)		
	Support referral		
	Care coordination		
	Trigger further discussion (*n* = 3)	Facilitates clinical discussions, particularly in opportunities for shared decision making with patients	
	Supported shared decision making (*n* = 2)		
	Communicating with patients		
	Patient satisfaction		
	Facilitate own judgment	Reminders improved clinician judgment and motivation, to provide recommended care and avoid dangerous situations	
	Remembering recommended orders		
	Good reminder		
	Increased motivation		
	Avoid dangerous situations		
	New knowledge (*n* = 2)	Useful sources of knowledge and advice during the consult	
	Useful sources of advice		
	Influenced treatment

**Table 3:** User barriers and enablers

	Finding	Category	Synthesised finding
**User barriers**	Lack of awareness (*n* = 2)	Not aware or does not see a need for tool	Providers experienced user barriers where awareness or need for the tool was limited, where the imposition of external authority through the CDS was unwelcome, and where users were resistant to technology
	Did not see a need		
	Providers hesitant		
	Perceived external authority	Users feeling marked down by an external authority	
	Being marked down		
	High-frequency overriders		
	Resistance to technology	Lack of trust or familiarity with technology	
	AI does not understand their jobs		
**User enablers**	Skill expansion (*n* = 2)	Perceived usefulness to expand skills	Providers experienced user enablers where CDS was seen as useful for skill expansion, where they felt confident with use of the tool following appropriate training and individual follow-up
	Usefulness (*n* = 2)		
	User satisfaction		
	Introductory training	Receiving initial and follow-up training	
	Following up session		
	Performance feedback		
	Confidence	Familiarity with tool	
	Familiarity

**Table 4:** External context barriers and enablers

	Finding	Category	Synthesised finding
**External context barriers**	Time-consuming (*n* = 6)	Additional time required or timing was not right	Providers experienced external context barriers where CDS was time-consuming and interrupted workflow without investment of additional resources, and where CDS use was limited by external upstream and downstream barriers
	Timing not right		
	Interruption to workflow (*n* = 2)	Disrupted and did not integrate with usual workflow	
	Difficult to integrate		
	Extra work		
	Financial incentives	Financial and resource limitations	
	Insufficient remuneration		
	Resource limitation		
	Levels of governance	Upstream and downstream barriers to using and following CDS recommendations	
	Downstream barriers		
	Challenge in following recommendations		
**External context enablers**	Engaged the principal [GP]	Leadership and allocated person to oversee CDS implementation	Providers experienced external context enablers where key personnel and teams were engaged, where use of the tool was easy and backed by technical support
	Screening champion		
	Allocated person		
	Team work	Engaging the team and maintaining staff skills	
	Maintaining staff skills		
	Easy to implement	Saving time and easy to implement	
	Saved time		
	Technical support	Providing technical support

**Table 5:** Technical barriers and enablers

	Finding	Category	Synthesised finding
**Technical barriers**	Not integrated into the EHR (*n* = 2)	Lack of integration and reliance on manual data collection	Providers experienced technical barriers where EHR integration was poor, where CDS displayed an excess number of prompts or had glitches
	Laborious data collection		
	Reliance on data		
	Lack of learning capacity of the system		
	Cluttered with stuff (*n* = 2)	Too much information or too many alerts	
	Burdensome prompts		
	Problem of multiple pop-ups		
	Glitches	Glitches and inaccuracies with alerts	
	Wrong alerts		
**Technical enablers**	Attractive design features (*n* = 2)	Attractive visuals and use of colour	Providers experienced technical enablers with attractive CDS designs, point of care availability of relevant information including historic data, where systems were easy to use and reliable, and had tailored functionalities
	Use of colour (*n* = 2)		
	Visual aide		
	Hands-on information	Patient information is immediately available at point of care	
	At a glance		
	Immediately there		
	Historical data	Information includes historical data	
	Faster than going to the files		
	Space saving		
	Ease of use	System is easy to use and reliable	
	Reliability is key		
	Drill-down functionality	Functionalities to examine population and individual level data	
	Identify and understand subgroups		

#### Clinical context barriers and enablers

“Clinical context” referred to clinical factors during the consultation—relating to the patient’s medical condition, patient-clinician interaction, or the clinician’s management of the patient. CDS barriers encountered during the consultation included “distorted priorities” and “blanket recommendations” that did not account for the patient’s agenda and the need to consider multiple clinical guidelines in multimorbidity presentations [74]. Enablers to CDS uptake included tools that facilitated structured chronic disease care or triggered relevant discussions during the clinical consultation. Clinicians also responded positively to CDS that facilitated their own judgment or provided a safety net to “avoid dangerous situations” [59].

Example finding 1: Distorted Priorities [74]

Illustration from interview: “One of the dangers I would see with this is the encouragement of game playing…So an electronic decision support module that is only related to cardiovascular disease…could lead you to focus on getting cholesterol and things done and perhaps forget immunisations or pap smears or the housing forms because that’s what the computer is flashing up at you.”

### User barriers and enablers

“User” factors referred to the individual attributes of the provider. Barriers arose from low awareness and low familiarity with the CDS technology. Lack of trust was also a major barrier—some clinicians felt “marked down” by an external authority [46, 74]. Perceived usefulness to skill expansion and user confidence were facilitators to CDS use. Building user confidence required deliberate effort from the implementation team in providing both initial training and follow-up training or individualised support. As the research teams plays a central role in driving engagement, one study described a plateauing of CDS use post-trial [72].

Example finding 2: Confidence [75]

Illustration from interview: “First, we were afraid that there was the need to handle computers and touch screens, but later after training, we were able to understand it. After we did 1 or 2 tests, it became easy, and we can do it better now.”

### External context barriers and enablers

“External context” referred to service and other macro context factors. Lack of time, interruption to workflow, and lack of resources were key barriers to CDS uptake. Time and remuneration are tightly coupled in fee-for-service settings, such as private general practice clinics. One team sought to include CDS use as a billable item number on the fee-for-service schedule in Australia but was informed that payment for CDS use was not possible from a legislative point of view [46]. Healthcare providers identified engaged clinical champions and allocated personnel to operate the CDS, including allocated technical support, as key enablers to successful implementation.

Example finding 3: Allocated person [72]

Illustration from interview: “A good single person allocated, keep monitoring, keep going, these tools will be very, very good. Yeah.”

### Technical barriers and enablers

“Technical” refers to technical features that affected CDS uptake. The alert burden was a recurring theme and contributed to CDS prompts being overridden [50]. Although the CDS systems included in this study were all EHR-linked, poor integration and the need for additional manual data entry were nevertheless barriers to uptake. Useful technical functions and attractive CDS design features were enablers to uptake. Clinicians valued CDS functions that provided relevant and immediate “hands-on information” [59]. Appropriate use of colour elicited desirability and “almost an emotional response” to the CDS [72].

Example finding 4: Integration [46]

Illustration from interview: “Nothing from HealthTracker populated into the EMR; [only] the reverse occurred.”

## Discussion

### Main findings and recommendations

Our systematic review aimed to describe the experience of healthcare providers in implementing, using, evaluating, and sustaining CDS systems. The findings of this review predominantly outline factors influencing implementation and use, since few findings from included primary research studies addressed factors influencing the evaluation or sustainability of CDS projects. The eight synthesised findings from meta-aggregation of qualitative findings related to clinical context, user-related, external context, and technical factors that influenced CDS uptake. We found that these factors influencing CDS uptake were universal across the broad range of included CDS studies, regardless of CDS type (e.g. by disease focus), and shared across both high and lower middle-income settings. Our findings indicate that adequate attention and resourcing needs to be directed at each of these domains when undertaking a CDS project. We summarise actionable steps that can be taken to address the challenges in each of these domains in Table 6.

**Table 6:** Recommendations for practice and research

**1. Recommendations for practice**	
a. Clinical context factors	
• Implement CDS with the goal of enhancing clinical processes (e.g. structured chronic disease care) rather than replacing clinical judgment with “blanket recommendations”	
• Use CDS to trigger discussions, but recognise that consultation priority should be set by patients and clinicians	
b. User factors	
• Ensure users are familiar with the CDS – what it can do and how to use it – through initial training and follow-up sessions	
• Help users to see where CDS can assist them, rather than see the CDS as an unwelcome, competing authority	
c. External context	
• Design CDS appropriate for existing workflows to save time and avoid extra work	
• At the service-level, structure a team of key clinician leaders and allocated personnel who will support CDS implementation and ongoing use	
d. Technical	
• Utilise user experience (UX) principals to design visually attractive and easy to use user interfaces (e.g. less is more, avoid alert overload)	
• Integrate CDS with existing EHR in real time to minimise laborious data entry	
**2. Recommendations for research**	
• Healthcare providers are frustrated with the current generation of simplistic, often single chronic disease focussed CDS tools. Future research can explore how CDS design and workflow can be better built for multimorbidity – this should include multiple perspectives from clinicians, informaticians, and software developers on what is feasible and how to achieve it.	
• CDS assists rather than replaces the complexity of human clinician decisions. Therefore, even with advances in technology, CDS is likely to remain “imperfect” from the user’s perspective. Future CDS implementation research can explore strategies to explicitly address user expectations, and identify user-led solutions to optimise the clinical utility of imperfect CDS technology.	
• CDS projects may stall post-implementation and after research team support is withdrawn. More health service-level research needs to be conducted to explore optimal financial reimbursement policies to sustain CDS uptake in routine clinical settings.	

In the CDS literature, external context and user factors affecting uptake have remained relatively constant over time and across heterogenous CDS implementations [18, 25, 86]. In particular, the macro external contextual “barriers of insufficiency”—lack of funding, training, and supportive policy—have changed little since barriers to health information technology was first described in the 1960s [18, 87]. Several included studies illustrate the challenge of overcoming barriers of insufficiency beyond the project implementation phase. Patel et al. highlighted the critical role of the CDS research team in driving training and user engagement—the authors noted that when this support was withdrawn post-trial, CDS-related improvements in health outcomes plateaued [72]. In Abimbola et al., the research team sought government funding to reimburse clinicians for CDS use on the national fee-for-service Medicare Benefits Schedule (Australia) but were informed that “from a legislative viewpoint, MBS items can’t be attached to software” [46]. Although there has been examples of national-level financial incentives for EHR-based CDS uptake (e.g. in the USA) [88], the lack of clear macro-level CDS reimbursement strategies continues to impede its widespread uptake into routine clinical practice [89, 90].

In contrast to external context and user factors, the clinical and technological expectations we identified in this review are specific to our highly digitised era. The “five rights” of CDS interventions include providing the right information, to the right person, in the right format, through the right channel, at the right time [4]. Providing the “right information” is increasingly hard to achieve in the face of multimorbidity—whilst single-disease digitalised guidelines may be appropriate in acute settings (e.g. stroke assessment), we found that a lack of CDS algorithm complexity frustrates clinicians and limits CDS applicability in multimorbid, chronic disease settings [28]. Clinician expectations for what constitutes right EHR information has also evolved in an age where handheld devices synchronise instantaneously to the cloud and across multiple devices, healthcare providers now expect pertinent individual EHR data to be immediately integrated within CDS systems. Today’s CDS users also expect “right format” not only in terms of correct functionality, but also in terms of desirability. Design principles suggest that attractive designs “look easier to use… whether or not they actually are easier to use” [91]—although user experience (UX) is an established sector outside of medicine, such applications of these principles to CDS research remains preliminary in nature [92, 93].

Thus, our results suggest that an ideal chronic disease CDS system would be wide rather than narrow in clinical scope to reflect the complex nature of care in multimorbidity. For example, some authors have proposed problem-orientated patient summaries with decision support as a potential way to improve CDS usability in multimorbidity settings [94, 95]. Such broad-scope CDS interventions require improved clinical collaboration beyond that of specialty or disease-specific interests [28, 96]. More work also needs to be done to develop CDS systems that are both functional and attractive. These goals are conceptually obvious but difficult to achieve in practice. For example, a patient’s data is often distributed across “archipelagos” of EHR sources, which may not adhere to interoperability standards [90]—greater collaboration between EHR vendors and developers is needed to enable a greater variety of EHR data to be extracted, which would increase CDS algorithm complexity and improve clinician workflow [95, 97, 98].

### Comparison with previous studies

In terms of methodology, our review is most similar to the systematic review of CDS studies conducted by Miller et al. (year 2000 to 2013), who used an inductive approach to qualitative evidence synthesis—similar to the JBI meta-aggregation method we used. However, Miller et al. included a narrower scope than us; whereas we examined both barriers and enablers, their search strategy narrowed the article search to primary research describing barriers or problems with CDS interventions only [23]. More recently, two 2021 systematic reviews examined barriers and enablers to using CDS systems—one investigating CDS targeting medication use [24] and one focussing on alert-based CDS systems [26]. Both of these reviews synthesised qualitative findings using thematic analysis with a deductive approach, utilising the “Human, Organization, and Technology–Fit” (HOT–fit) framework [99]. Several barriers and enablers identified in our study are similar to the human, technological, and organisation factors influencing uptake described by the HOT-fit framework [86, 99, 100]. In one of these reviews, Westerbeek et al. highlighted that disease-specific factors were not adequately captured within the HOT-fit framework [24]. Likewise, we found a large number of qualitative findings relating to “clinical context” factors (e.g. distorted clinical priorities) that are not sufficiently incorporated into HOT-fit [99] and similar determinant frameworks used for health information technology interventions [25, 58, 86].

### Strengths and limitations

To date, the discourse on CDS systems implementation has typically focussed on barriers to uptake. This study takes a unique approach to synthesising provider experiences, focussing not only on deficits but also on potential solutions to improving CDS interventions [101]—specifically, our meta-aggregation process extracted and synthesised findings on healthcare provider perspectives of CDS enablers and presented them as actionable steps for practice. Another strength of our review was the breadth of primary research articles included to capture the experience of CDS interventions across different settings and clinical specialties. We utilised a broad search strategy for EHR-based CDS systems, across high- and lower middle-income countries, and across various non-acute clinical settings. To incorporate the spectrum of experience across both early and established CDS systems, we included qualitative research and “other evaluation” studies with qualitative findings. Even though studies in the other evaluation category tended to have limited methodological rigour, we included them to improve generalisability of our findings. We note that other evaluation studies with qualitative findings are often conducted as early precursors of formal qualitative studies, or are sometimes the only evaluations that took place if projects lacked sufficient sustainability to undertake formal qualitative evaluations.

There are several limitations to our review methodology. Whilst we employed a comprehensive search strategy across several databases, we did not include grey literature. Furthermore, non-research articles such as perspective pieces and systematic reviews were not included as the focus was on synthesis of primary research article findings. Common, related cardiometabolic chronic diseases with similar modifiable risk factors were included in this review—however, other chronic diseases such as COPD or chronic pain were not included. Healthcare providers are the primary targets of EHR-based CDS and thus were included as the population of interest in this study. However, multiple perspectives, including patient, EHR vendor, CDS developer, and other collaborator perspectives are also of interest in fully understanding the barriers and facilitators to CDS success [102, 103].

JBI methodology to meta-aggregation is one of many forms of qualitative research synthesis, and debates over the preferred systematic review synthesis methodology continue [34, 104]. Consistent with pragmatism, knowledge from meta-aggregation do not indicate hypothetical explanation [105] and some critics note a lack of re-interpretation and generation of theoretical understandings with this approach. We recognise the important contributions of theory and frameworks in the field of implementation research, which facilitates the systematic uptake of effective health technologies into routine clinical contexts [86, 106–108]. Diverse theoretical lenses have been used in describing CDS uptake [47, 100, 109, 110], and we provide a comprehensive description of theoretical frameworks used to guide qualitative data analysis within the primary research articles included in our review (e.g. NASSS framework, CFIR framework). However, because meta-aggregation is descriptive rather than interpretive, a limitation of this synthesis method is that it does not further develop existing theoretical frameworks. The merits of alternative qualitative synthesis approaches over meta-aggregation lie in their ability to contribute to mid-level theory—such alternative synthesis methods include meta-ethnography, or deductive thematic analysis using an existing implementation science framework. Nevertheless, meta-aggregation was selected as the most appropriate synthesis method for our review given the limited availability of “thick” and “rich” qualitative data [36, 37] in the CDS field. We also selected meta-aggregation because it is a structured and transparent method to synthesise findings into concrete statements (practice-level theory) that is practical and accessible to healthcare providers at the coalface of CDS implementation [42, 111].

## Conclusion

Qualitative findings of barriers and enablers to CDS uptake provide valuable insights into why some projects are successfully implemented, whereas others fail to achieve uptake. Our systematic review summarises provider experiences in implementing and using chronic disease CDS systems across a broad range of studies. Our findings identified clinical context, user, external context, and technological factors affecting uptake. The meta-aggregated findings and summary of recommendations provide an evidence-base for designing, implementing, and sustaining future CDS systems.

## Supplementary Information

**Additional file 1.** PRISMA 2020 Checklist.

**Additional file 2.** Search strategy.

**Additional file 3.** Detailed inclusion and exclusion criteria.

**Additional file 4.** Methodological quality of included studies.

**Additional file 5.** List of findings with illustrations.

## Data Availability

The data supporting the findings of this study are available within the article and Supplementary Information.

## Abbreviations

AF: Atrial fibrillation
CDS: Clinical decision support
CeHRes: Centre for eHealth Research (CeHRes) Roadmap
CFIR: Consolidated Framework for Implementation Research
CKD: Chronic kidney disease
CVRF: Cardiovascular risk factors
EHR: Electronic health record
GP: General practitioners
HITREF: Health Information Technology Evaluation Framework
HOT-fit: Human, Organization, and Technology–Fit framework
JBI: Formerly Joanna Briggs Institute
JBI SUMARI: JBI System for the Unified Management, Assessment and Review of Information
NASSS: Nonadoption, Abandonment, and challenges to Scale-up, Spread and Sustainability framework
NPT: Normalisation Process Theory
PRISMA: Preferred Reporting Items for Systematic reviews and Meta-Analyses
UX: User experience
