# Supplementary material for: Barriers and enablers to implementing and using clinical decision support systems for chronic diseases: a qualitative systematic review and meta-aggregation
Source: Implement Sci Commun. 2022 Jul 28;3:81. doi: 10.1186/s43058-022-00326-x (PMC9330991; doi:10.1186/s43058-022-00326-x)
Supplement: Supplementary file 2 — Additional file 2. Search strategy. [file 43058_2022_326_MOESM2_ESM.pdf]

Additional file 2 – Search strategy

Searches were conducted in PubMed (Database: Medline), EBSCOHOST (Databases: CINAHL, APA PsychInfo, EconLit) and Web of Science. Full search strategy for PubMed is outlined below.

	**PubMed (Database: Medline)**
#1	"decision support"[tiab] OR "decision support systems, clinical"[mh] OR "therapy, computer-assisted"[mh:noexp] OR "reminder systems"[mh] OR "drug therapy, computer-assisted"[mh] OR "medical order entry systems"[mh] OR "Decision Making, Computer Assisted"[mh:noexp]
#2	((computer*[tiab] OR electronic[tiab]) AND (alert*[tiab] OR reminder*[tiab] OR recommendation*[tiab] OR dashboard[tiab] OR "order set" OR "order sets" OR guideline*))
#3	(randomized[tiab] AND reminder*[tiab]) OR (randomised [tiab] AND reminder* [tiab])
#4	(cpoe[tiab] OR "physician order entry"[tiab] OR "provider order entry"[tiab] OR "clinical decision support system"[tiab] OR "clinical decision support systems"[tiab])
#5	#1 or #2 or #3 or #4
#6	“kidney diseases”[mh] OR “renal replacement therapy”[mh] OR “renal insufficiency”[mh:noexp] OR “renal insufficiency, chronic”[mh]
#7	“kidney disease*”[tiab] OR “renal disease*”[tiab] or “kidney failure”[tiab] or “renal failure”[tiab] or ESRF[tiab] or ESKF[tiab] or ESRD[tiab] or ESKD[tiab] or CKF[tiab] or CKD[tiab] or CRF[tiab] or CRD[tiab]
#8	predialysis[tiab] OR “pre-dialysis”[tiab] OR dialysis[tiab] OR hemodialysis[tiab] OR haemodialysis[tiab] OR CAPD[tiab]
#9	#6 OR #7 OR #8
#10	“diabetes mellitus”[mh] OR “diabetes mellitus”[tiab] OR “DM”[tiab] OR diabetes[tiab] OR T2DM [tiab] OR T1DM[tiab]
#11	hyperglycaemia[mh] OR hyperglycaemia[tiab] OR hyperglycemia[tiab] OR “pre-diabet*”[tiab] OR “prediabet*”[tiab] OR “high blood sugar”[tiab] OR “gestational diabetes”[tiab] OR “diabetes, gestational”[mh:noexp]
#12	#10 OR #11
#13	hypertension[mh] OR hypertension[tiab] OR hypertensive[tiab] OR “hypertens*”[tiab] OR “blood pressure”[tiab] OR SBP[tiab] OR DBP[tiab]
#14	dyslipidemias[mh] OR dyslipidemia*[tiab] OR dyslipidaemia*[tiab] OR hypercholesterol*[tiab] OR hyperlipid*[tiab] OR “high cholesterol”[tiab]
#15	#13 OR #14
#16	“cardiovascular diseases”[mh] OR “cardiovascular disease*”[tiab] OR “cardiovascular disorder*”[tiab] OR “heart disease”[tiab]
#17	“peripheral vascular disorder*”[tiab] OR “peripheral vascular disease*”[tiab] OR “heart failure”[tiab] OR "cardiac failure"[tiab] OR "congestive heart failure"[tiab] OR “coronary artery disease”[tiab] OR “coronary arterial disease”[tiab] OR “coronary heart disease”[tiab] OR “stroke”[tiab] OR “cerebrovascular event”[tiab] OR “cerebrovascular accident”[tiab] OR “cerebrovascular disease”[tiab] OR “heart attack”[tiab] OR “ischaemic heart disease”[tiab] OR “myocardial infarction”[tiab]
#18	#16 or #17
#19	#9 OR #12 OR #15 OR #18
#20	#5 and #19
#21	Filters English, Human
#22	2011/01:2021/01[dp]
