# Supplementary material for: Barriers and enablers to implementing and using clinical decision support systems for chronic diseases: a qualitative systematic review and meta-aggregation
Source: Implement Sci Commun. 2022 Jul 28;3:81. doi: 10.1186/s43058-022-00326-x (PMC9330991; doi:10.1186/s43058-022-00326-x)
Supplement: Supplementary file 3 — Additional file 3. Detailed inclusion and exclusion criteria. [file 43058_2022_326_MOESM3_ESM.pdf]

# Additional file 3: Detailed inclusion and exclusion criteria

The detailed inclusion and exclusion criteria listed here is for the overall CDS systematic review, a mixed-method review incorporating qualitative, effectiveness, and economic evaluation components. Only the qualitative outcomes in the inclusion criteria apply to this study.

| Inclusion criteria |
|---|
| **Population:** CDS must be for adults (>18 years) receiving chronic disease care:<br>1. Chronic kidney disease: including monitoring of acute kidney injury in community settings.<br>2. Cardiovascular disease: including ischaemic heart disease, cerebrovascular disease, peripheral vascular disease, and abdominal aortic aneurysm. Includes diagnosis of chest pain and transient ischaemic attack in community settings.<br>3. Diabetes: including type 1 diabetes mellitus, type 2 diabetes mellitus, gestational diabetes mellitus.<br>4. Hypertension<br>5. Hypercholesterolaemia<br>6. Other cardiovascular risk factors: metabolic syndrome, obesity. |
| **Intervention:** EHR-enabled, computerised CDS used in any non-acute setting. CDS includes and is not limited to:<br>1. CDS targeting chronic disease care: for screening, diagnosis, pathway support, pharmacological management, non-pharmacological management.<br>2. CDS functions: including tools for clinical documentation, data presentation, order or prescription creation, protocol or pathway support, reference guidance, and alert or reminder.<br>3. CDS used in any setting for chronic disease care: including primary care, specialist outpatient, and community health services.<br>May include registries and other databases derived from EHR if the system includes an EHR-based CDS. |
| **Comparison:** Comparison can be a control group receiving placebo or standard care, or a historic control for effectiveness outcome studies only. |
| **Outcomes:** Outcomes include:<br>1. Effectiveness outcomes: including clinical effectiveness (e.g. morbidity, mortality), health process outcomes (e.g. adherence to guidelines), workload and efficiency outcomes (e.g. time saved).<br>2. Economic outcomes: including cost, cost-effectiveness, cost-utility, cost-benefit outcomes.<br>3. Qualitative outcomes: experience of healthcare providers (individual clinicians or services) in implementing, using, evaluation and sustaining CDS interventions. |
| **Other eligibility criteria:** Time restricted to publications from January 2011 to January 2021. English language studies only. |

| Exclusion criteria |
|---|
| **Population:** CDS for paediatric populations (<18 years). CDS were excluded if they targeted patients:<br>1. With acute conditions: e.g. deep vein thrombosis, pulmonary embolism, acute kidney injury in hospital setting, communicable diseases.<br>2. Within acute settings: e.g. intensive care unit, surgical, inpatient settings. |
| **Intervention:** CDS interventions were excluded if they were: |

| |
|---|
| 1. CDS without an EHR component: e.g. manual paper-based CDS without an EHR component, CDS in a stand-alone web-based application, CDS as a component of patient education module without links to EHR records, telehealth with CDS component without link to EHR.<br>2. EHR without a CDS intervention: e.g. implementation of new EHR.<br>3. Electronic monitoring interventions without an EHR component: e.g. biosensor or biological monitoring, robotics, ECG monitoring.<br>4. CDS with limited scope for chronic disease care: e.g. sole focus on image interpretation (CT diagnoses, retinal screening), sole focus on single medication dosing (insulin, warfarin dosing), simple alert or reminder (EHR-generated patient recall alerts). |
| **Comparison:** Studies without a control group for effectiveness outcomes only. |
| **Outcomes:** No exclusions based on outcome. |
| **Other exclusion criteria:** Excluded studies:<br>1. Publication: before 2011, non-English language.<br>2. Article type: not primary research articles (e.g. perspective pieces, systematic reviews, editorials), or were protocols of primary research studies only.<br>3. CDS development or system in test conditions only: e.g. modelling or technical methods–risk prediction, AI data mining, model performance, ontology development.<br>4. CDS testing in test conditions only: prototype not used in the clinical setting. |

**Abbreviations:**

AI – Artificial intelligence

CDS – Clinical decision support

ECG – Electrocardiogram

EHR – Electronic health record
