# Supplementary material for: Barriers and enablers to implementing and using clinical decision support systems for chronic diseases: a qualitative systematic review and meta-aggregation
Source: Implement Sci Commun. 2022 Jul 28;3:81. doi: 10.1186/s43058-022-00326-x (PMC9330991; doi:10.1186/s43058-022-00326-x)
Supplement: Supplementary file 4 — Additional file 4. Methodological quality of included studies. [file 43058_2022_326_MOESM4_ESM.pdf]

Additional file 4: Methodological quality of included studies

The "JBI critical appraisal checklist for qualitative research (2020)" from the JBI Manual of Evidence Synthesis (1) was used for this systematic review. Responses are coded as yes (Y), no (N), or unclear (U). For qualitative studies all questions in the checklist were used. For "other evaluation studies" with qualitative findings, Q1 to Q7 were considered not applicable (N/A).*

*For the purposes of this review "qualitative studies" referred to studies with an explicit qualitative methodological approach (e.g. phenomenology). "Other evaluation studies" refers to studies incorporating a qualitative inquiry component (e.g. user feedback study) but without an explicit qualitative methodological approach.

Table 1: Studies with qualitative findings

Citation	Q1	Q2	Q3	Q4	Q5	Q6	Q7	Q8	Q9	Q10
Abimbola S, Patel B, Peiris D, Patel A, Harris M, Usherwood T, et al. 2019.	Y	Y	Y	Y	Y	Y	N	Y	N/A	Y
Ballard AY, Kessler M, Scheitel M, Montori VM, Chaudhry R. 2017.	N/A	N/A	N/A	N/A	N/A	N/A	N/A	Y	Y	Y
Chiang J, Furler J, Boyle D, Clark M, Manski-Nankervis JA. 2017.	N/A	N/A	N/A	N/A	N/A	N/A	N/A	Y	Y	Y
Cho I, Slight SP, Nanji KC, Seger DL, Maniam N, Dykes PC, et al. 2014.	N/A	N/A	N/A	N/A	N/A	N/A	N/A	U	Y	Y
Conway N, Adamson KA, Cunningham SG, Emslie Smith A, Nyberg P, Smith BH, et al. 2018.	N/A	N/A	N/A	N/A	N/A	N/A	N/A	N	N/A	U
Dagliati A, Sacchi L, Tibollo V, Cogni G, Teliti M, Martinez-Millana A, et al. 2018.	N/A	N/A	N/A	N/A	N/A	N/A	N/A	N	Y	Y
Dixon BE, Alzeer AH, Phillips EO, Marrero DG. 2016.	N/A	N/A	N/A	N/A	N/A	N/A	N/A	N	Y	Y
Fico G, Hernanzez L, Cancela J, Dagliati A, Sacchi L, Martinez-Millana A, et al. 2019.	Y	Y	Y	U	Y	N	N	N	Y	Y
Gill J, Kucharski K, Turk B, Pan C, Wei W. 2019.	N/A	N/A	N/A	N/A	N/A	N/A	N/A	N	Y	U

Gold R, Bunce A, Cowburn S, Davis JV, Nelson JC, Nelson CA, et al. 2019.	U	Y	Y	N	U	N	N	N	Y	Y
Helldén A, Al-Aieshy F, Bastholm-Rahmner P, Bergman U, Gustafsson LL, Höök H, et al. 2015.	N/A	N/A	N/A	N/A	N/A	N/A	N/A	Y	N	Y
Holt TA, Dalton AR, Kirkpatrick S, Hislop J, Marshall T, Fay M, et al. 2018.	Y	Y	Y	Y	Y	N	N	Y	Y	Y
Jindal D, Gupta P, Jha D, Ajay VS, Goenka S, Jacob P, et al. 2018.	N/A	N/A	N/A	N/A	N/A	N/A	N/A	Y	N	Y
Kumar S, Woodward-Kron R, Frank O, Knieriemen A, Lau P. 2018.	U	U	Y	Y	Y	N	N	Y	Y	Y
Litvin CB, Hyer JM, Ornstein SM. 2016.	N/A	N/A	N/A	N/A	N/A	N/A	N/A	Y	Y	Y
Lopez PM, Divney A, Goldfeld K, Zanowiak J, Gore R, Kumar R, et al.	N/A	N/A	N/A	N/A	N/A	N/A	N/A	N	N	U
Lugtenberg M, Pasveer D, van der Weijden T, Westert GP, Kool RB. 2015.	N/A	N/A	N/A	N/A	N/A	N/A	N/A	Y	N/A	Y
Majka DS, Lee JY, Peprah YA, Lipiszko D, Friesema E, Ruderman EM, et al. 2019.	N/A	N/A	N/A	N/A	N/A	N/A	N/A	N	Y	U
Meador M, Osheroff JA, Reisler B. 2018.	N/A	N/A	N/A	N/A	N/A	N/A	N/A	U	N/A	Y
Millery M, Shelley D, Wu D, Ferrari P, Tseng TY, Kopal H. 2011.	U	Y	Y	U	U	N	N	Y	Y	Y
O'Reilly DJ, Bowen JM, Sebaldt RJ, Petrie A, Hopkins RB, Assasi N, et al. 2014.	N/A	N/A	N/A	N/A	N/A	N/A	N/A	N	N	U
Orchard J, Li J, Gallagher R, Freedman B, Lowres N, Neubeck L. 2019.	Y	Y	Y	Y	Y	N	N	Y	Y	Y

Patel B, Usherwood T, Harris M, Patel A, Panaretto K, Zwar N, et al. 2018.	Y	Y	Y	Y	Y	N	N	Y	Y	Y
Peiris D, Usherwood T, Weeramanthri T, Cass A, Patel A. 2011.	Y	Y	Y	Y	Y	Y	Y	Y	Y	Y
Praveen D, Patel A, Raghu A, Clifford GD, Maulik PK, Abdul AM, et al. 2014.	Y	Y	Y	Y	Y	N	N	Y	Y	Y
Raghu A, Praveen D, Peiris D, Tarassenko L, Clifford G. 2015.	N/A	N/A	N/A	N/A	N/A	N/A	N/A	N	Y	U
Regan ME. 2017.	N/A	N/A	N/A	N/A	N/A	N/A	N/A	N	N	U
Romero-Brufau S, Wyatt KD, Boyum P, Mickelson M, Moore M, Cognetta-Rieke C. 2020.	N/A	N/A	N/A	N/A	N/A	N/A	N/A	N	N	U
Shemeikka T, Bastholm-Rahmner P, Elinder CG, Vég A, Törnqvist E, Cornelius B, et al. 2015.	N/A	N/A	N/A	N/A	N/A	N/A	N/A	N	N	U
Singh K, Johnson L, Devarajan R, Shivashankar R, Sharma P, Kondal D, et al. 2018.	Y	Y	Y	Y	Y	N	N	Y	Y	Y
Sperl-Hillen JM, Crain AL, Margolis KL, Ekstrom HL, Appana D, Amundson G, et al. 2018.	N/A	N/A	N/A	N/A	N/A	N/A	N/A	N	Y	U
Vedanthan R, Blank E, Tuikong N, Kamano J, Misoi L, Tulienge D, et al. 2015.	U	U	Y	Y	Y	N	N	Y	N	Y
Wan Q, Makeham M, Zwar NA, Petche S. 2012.	U	U	Y	Y	Y	N	N	Y	Y	Y
**%**	**24.24**	**30.3**	**39.39**	**30.3**	**33.33**	**6.06**	**3.03**	**51.51**	**63.63**	**69.69**

References

1. Aromataris E, Munn Z. JBI Manual for Evidence Synthesis: JBI; 2020 [cited 2021 November]. Available from: https://synthesismanual.jbi.global.
