# Supplementary material for: Barriers and enablers to implementing and using clinical decision support systems for chronic diseases: a qualitative systematic review and meta-aggregation
Source: Implement Sci Commun. 2022 Jul 28;3:81. doi: 10.1186/s43058-022-00326-x (PMC9330991; doi:10.1186/s43058-022-00326-x)
Supplement: Supplementary file 5 — Additional file 5. List of findings with illustrations. [file 43058_2022_326_MOESM5_ESM.pdf]

Additional file 5: List of findings with illustrations

As per JBI Manual for Evidence Synthesis, unequivocal (U), credible (C), and not supported (N) are defined as:

Unequivocal: Findings accompanied by an illustration that is beyond reasonable doubt and; therefore not open to challenge.

Credible Findings accompanied by an illustration lacking clear association with it and therefore open to challenge.

Not Supported: Findings are not supported by the data.

**Study: Abimbola et al, 2019**	
Finding	Attractive design features (U)
Illustration	Interview: "loved the traffic light... loved seeing the graphs"
Finding	Structured care (U)
Illustration	Interview: "telling me what I needed to do to ensure that... everything's been covered"
Finding	Glitches (U)
Illustration	Interview: "it's never worked"
Finding	Integration (U)
Illustration	Interview: “nothing from HealthTracker populated into the EMR; [only] the reverse occurred”
Finding	Being marked down (U)
Illustration	Interview: “don’t tell me to do something when I’ve made an active decision in discussion with my patient to not do it, don’t keep giving me a red traffic light”
Finding	Levels of governance (C)
Illustration	Interview: “they weren’t given the green light by the head of the board”
Finding	Engaged the principal [GP] (C)
Illustration	Interview: “once you’ve engaged the principal or principals, and if they’re taken with the idea, then they’ll just do it”
Finding	Financial incentives (U)
Illustration	Interview: “we put in a submission to the federal government only to be told eventually that from a legislative viewpoint, MBS items can’t be attached to software”
Finding	Maintaining staff skills (C)
Illustration	Interview: "someone on the ground who is familiar with the tool inside out"

Finding	Number of conditions (C)
Illustration	Interview: "if it could be developed for a whole range of interventions that might be sustainable"

**Study: Ballard et al, 2017**	
Finding	Lack of awareness (C)
Illustration	Survey free text: “Often forget it exists”
Finding	Lack of applicability (C)
Illustration	Survey free text: “Most of my patients are already on insulin and/or oral agents”
Finding	Time constraint (C)
Illustration	Survey free text: “Computer in rooms often do not pull up the decision aid fast enough”
Finding	Impacts treatment decision (N)
Illustration	Survey: Impacts treatment decision 22-42% of the time on survey results

**Study: Chiang et al, 2017**	
Finding	Share decision-making (C)
Illustration	Interview: "I think it would help people become motivated to make change"
Finding	Cluttered with stuff (U)
Illustration	Interview: "I think the hardest thing with the pop-up tool is actually getting the balance right, so you’re not getting too many that it is really cluttered with stuff you don’t need to read"
Finding	Technical support (C)
Illustration	Interview: "we have to have really good support with both ends of the developer"

**Study: Cho et al, 2014**	
Finding	High-frequency overriders (C)
Illustration	Authors: Among the alerts, 4,120 were renal alerts and were triggered by 584 prescribers (34% of all prescribers), and 3,221 (78.2%) were overridden

**Study: Conway et al, 2018**	
Finding	Relevance (N)

Illustration	Authors: majority of respondents had a positive or neutral response to the content of the reminders
Finding	System use (N)
Illustration	Authors: self-reported use of the system was low
Finding	Performance expectancy (N)
Illustration	Authors: significantly higher for nurses
Finding	User fatigue (N)
Illustration	Authors: concerns regarding user fatigue
Finding	Workflow (N)
Illustration	Authors: some users using the system within their normal clinical workflow to improve the efficiency of their use

**Study: Dagliati et al, 2018**	
Finding	Drill-down functionality (U)
Illustration	Focus group: drill-down functionality allows identification of subjects not compliant with guidelines and inspection of their individual data
Finding	Identify and understand subgroups (C)
Illustration	Focus group: identify and understand the characteristics of subgroups of patients (temporal phenotypes)

**Study: Dixon et al, 2016**	
Finding	Quality (N)
Illustration	Survey: "not confident" of CDS info
Finding	System use (N)
Illustration	Survey: 66% reported using at least once during pilot
Finding	Technology (N)
Illustration	Authors: technology not easy to use in workplace
Finding	Usefulness (N)
Illustration	Authors: patients "always bring their pill bottles" for independent verification regardless of CDS

**Study: Fico et al, 2019**	
Finding	User satisfaction (C)

Illustration	Survey: “almost very good” for... pragmatic quality, stimulation and identification, and attractiveness
Finding	Usability (N)
Illustration	Authors: need of a technical person to be able to use the system, the perception of the system as cumbersome and the necessity to learn too many things before using the system
Finding	Effort (N)
Illustration	Authors: breaking and disruptive routine and therefore more work and efforts in terms of user training, and set up of organizational and procedural measures
Finding	Functionalities (N)
Illustration	Authors: refine tuning features for customized filtering and temporal analyses... personalize the display of the results
Study: Gill et al, 2019	
Finding	Reimbursement (N)
Illustration	Interview: insurers not paying for the staff and time required to implement team-based care
Finding	Improve quality of care (N)
Illustration	Interview: participants agreed CDS and patient-centred medical home (PCMH) were important in improving quality of care
Finding	Inaccuracy (N)
Illustration	Interview: inaccuracy of alerts (eg, lack of records of testing performed by specialists, such as endocrinologists) or inaccuracies in the communication of data between the EHR and the CDS

**Study: Gold et al, 2019**	
Finding	Accuracy (N)
Illustration	Authors: tools’ accuracy was questioned... Given these issues, some clinic leadership or staff felt the tools were not worth promoting.
Finding	Complex (N)
Illustration	Authors: study clinic staff found implementation toolkit complex
Finding	Follow-up (N)
Illustration	Authors: staff reported that their qualitative team check-ins kept targeted outcomes on their radar
Finding	Applicability (N)

Illustration	Authors: clinic staff generally did not focus on improving ACE/ARB prescribing
Finding	Practice requirements (N)
Illustration	Authors: not perfectly compatible with practice requirements, as some of its content did not align with quality measures that the study CHCs had to address in value-based payment structures

**Study: Hellden et al, 2015**	
Finding	Hands-on information (U)
Illustration	Focus group: "The individual adjustments of dosages were used in greater extent since the GFR value was given automatically and together with advice on dosages."
Finding	Knowledge (C)
Illustration	Focus group: "Old knowledge became more available with the new technique. The renal button and the interaction service had reciprocal positive effects on each other."
Finding	Workflow (C)
Illustration	Focus group: "The “renal button” gave a certain amount of more work, but it was positive because it facilitated the judgment, medication, and increased safety."
Finding	Avoid dangerous situations (C)
Illustration	Focus group: "You read, correct, and work more preventively, and avoid dangerous situations that could have meant admission to hospital."
Finding	Facilitate own judgment (U)
Illustration	Focus group: "The advice should not be directive but give attention so that they facilitate your own judgment."
Finding	Colour (C)
Illustration	Focus group: "The colour marking should remain also after the dose adjustment in order to mark out the patient’s renal function."
Finding	Not integrated into the EHR (U)
Illustration	Focus group: "It is a shortcoming that APO-doses (pre-dispensed medication) are not integrated into the EHR computer record."

**Study: Holt et al, 2018**	
Finding	Trigger further discussion (U)

Illustration	Interview: "I think it's very useful to remind us … because sometimes patients change their attitudes, and this does help to remind you of that, you must have that discussion again with some patients"
Finding	Useful (C)
Illustration	Interview: "For some of the other partners, I have to say, who are reluctant themselves to put elderly patients on anticoagulants for perceived risks … it's been helpful for them as well"
Finding	Own decisions (N)
Illustration	Interview: "I can make recommendations and they can ask me my opinion … but people take time to come to their own decisions"
Finding	Problem of multiple pop-ups (C)
Illustration	Interview: 'We might have six or seven of them [reminders], and once you've… worked through about three or four... you think, well that's another 5 or 10 minutes of consultation… so you leave it.'
Finding	Patient's own agenda (C)
Illustration	Interview: "It really depended on the patient's agenda, you don't want to make them feel they haven't actually had the outcome they wanted from the consultation before you dive in and actually talk about what you want to talk about"
Finding	Timing not right (C)
Illustration	Interview: "If they come and they're actually sick, you can't then talk to them about a preventative medication because, you know, they're too unwell to start it anyway."

**Study: Jindal et al, 2018**	
Finding	Useful (N)
Illustration	Interview: "Yes it is helpful because we treat patients in a different way now, so patients feel that staff are more responsible and working hard"
Finding	Skills improved (U)
Illustration	Interview: "My working skills have improved because previously I never used to work like this. mWellcare has helped me to learn new things"
Finding	At a glance (C)
Illustration	Interview: "It has improved the quality of patient assessment, as it provides the patient summary at a glance in the DSR print out"
Finding	Challenge in following recommendations (C)
Illustration	Interview: "the unavailability of the drug is the major challenge in following the recommendations"

Finding	Cluttered (U)
Illustration	Interview: "the DSR output is cluttered and the name of the medicines is mentioned as the class name so we find it difficult in thinking of the generic name"
Finding	Time-consuming (C)
Illustration	Interview: "Patient assessment through this is time-consuming; sometimes patients get irritated and we have long queues"

**Study: Kumar et al, 2018**	
Finding	Prioritisation (C)
Illustration	Interview: "A lot of the doctors will gloss over the prompts on the computer because they don't see them or they don't respond to prompts or um … they're busy. So, a prompt from the patient is gonna be acted on more likely than what's on the software."
Finding	Pressure of time (C)
Illustration	Interview: "I mean the issue then becomes how do you fit it all into the consult? Which is always a concern…"
Finding	Health literacy (U)
Illustration	Interview: "People have to have sufficient education. So it has to do with health literacy really."
Finding	Reliance on data (C)
Illustration	Interview: "Sometimes it's as little an issue as where the information has been entered and whether it's been extracted correctly."
Finding	Interruption to workflow (C)
Illustration	Interview: "the reception staff had to leave their desk and move two or three paces aside [to get the reminder from the printer], and that wasn't always practical."
Finding	Access (N)
Illustration	Interview: "It's giving access to what's on the database or what isn't there… when you're busy in general practice, it's not always easy to see."
Finding	Insufficient remuneration (U)
Illustration	Interview: "it takes a lot of time. And, you know, we're not well remunerated for that."
Finding	Complexity of chronic disease (N)
Illustration	Interview: "You look at swiss-cheese theory errors made by people. The errors aren't the individuals, there's the system itself."

**Study: Litvin et al, 2016**	
Finding	Attention (N)
Illustration	Authors: CDS helps focus provider attention on CKD
Finding	Disagreement (N)
Illustration	Authors: some providers did not agree with monitoring albuminuria in patients already taking an ACEI or ARB
Finding	Troubleshoot (N)
Illustration	Authors: At both site visits, the research team was able to make minor tweaks to the CDS tools to resolve any issues and ensure that they best accommodated practice workflow.
Finding	Extra clicks (N)
Illustration	Authors: CDS required extra clicks
Finding	Capture (N)
Illustration	Authors: did not capture labs ordered by specialists
Finding	Overdiagnosing (N)
Illustration	Authors: some providers noted concern about overdiagnosing patients with CKD and also reported that patients were concerned when seeing this diagnosis in an after-visit summary
Finding	Forgetting (N)
Illustration	Authors: Some providers also reported overlooking some of the CDS tools available to them... many providers reported forgetting that it was available.

**Study: Lopez et al, 2019**	
Finding	Registry reports (N)
Illustration	Authors: registry reports helped them to reliably see which of their patients had uncontrolled hypertension and prompted them to call patients to schedule a clinic visit
Finding	Easy to implement (C)
Illustration	Interview: [alerts and order sets were] “easy to implement”
Finding	Monitor (N)
Illustration	Authors: improved their ability to monitor and serve at-risk patients
Finding	Limited time (N)
Illustration	Authors: limited time and high staff turnover slowed technical advancement to navigate new features within an EHR system

Study: Lugtenberg et al, 2015	
Finding	Rarely use the system (N)
Illustration	Survey: half of the GPs (52 %) and 42 % of the PNs reported to either never or rarely use the system
Finding	Useful sources of advice (C)
Illustration	Survey: 80 % of the GPs and 67 % of the PNs agreed to the statement that CDSSs are useful sources of advice
Finding	Difficult to integrate (C)
Illustration	Survey: Sixty percent of the GPs and 25 % of the PNs indicated that the use of CDSSs is difficult to integrate in daily practice.
Finding	Not aware (C)
Illustration	Survey: 2% of the GPs and 18 % of the PNs were not aware of the existence of NHGDoc at all
Finding	Cookbook medicine (C)
Illustration	Survey: About a third of both groups (36 % of GPs and 31 % of PNs) was afraid that using CDSSs leads to ‘cookbook medicine’.
Finding	Lack of learning capacity of the system (U)
Illustration	Survey: In both groups a lack of learning capacity of the system (80 and 75 %)... and a lack of responsiveness of the system (33 and 38 %) were often perceived as barriers.
Finding	Limited time (C)
Illustration	Survey: Sixty percent of the GPs indicated the limited time available to be a barrier, whereas only 16 % of the PNs considered this to be a barrier.
Finding	Extra work (C)
Illustration	Survey: 60 % of GPs reported that using the CDSS required too much extra work, as compared to 27 % of the PNs.
Finding	Negative effect on communication (C)
Illustration	Survey: From the patient-related factors, particularly a negative effect on patient communication was considered as a barrier by 26 % of the GPs and 22 % of the PNs.

Study: Majka et al, 2019	
Finding	Risk factor measurement (N)
Illustration	Survey: Rheumatology providers expressed approval of the EHR tools used to improve risk factor measurement and referral to primary care providers.
Finding	Improved quality of care (C)

Illustration	Survey: EHR tools improved quality of care - streamlined electronic order-sets (100%)
Finding	Support referral (N)
Illustration	Survey: support automatic direct referral to internal medicine on my behalf for patients with uncontrolled CVD risk factors (92%)
Finding	Personalized (N)
Illustration	Survey: would like more patients with uncontrolled CVD risk factors to receive personalized risk reports (83%)

**Study: Meador et al, 2018**	
Finding	Standardized (C)
Illustration	Interview: “The algorithms are helpful because previously each provider utilized a different process of identification.”
Finding	Providers hesitant (U)
Illustration	Interview: “there will always be some patients that meet the hypertension criteria but providers do not wish to diagnose them for whatever reason.”
Finding	Impact (N)
Illustration	Interview: “We are in the beginning stages of implementation, so there has not been enough data to determine the effects of the protocol/program.”
Finding	Resource limitation (U)
Illustration	Interview: “Finding the time to perform all the necessary evaluation steps and compliance. Providing additional training of staff is difficult with the limited resources.”

**Study: Millery et al, 2011**	
Finding	Perceived usefulness (N)
Illustration	Interview: "The alerts are superb"
Finding	Remembering recommended orders (C)
Illustration	Interview: “I like to be validated in what I do, and since this is not my typical patient that I see, I like to see that. The little hint for the labs, the immunizations, and the appointments are all the pros of it.”
Finding	Reminders algorithm (N)
Illustration	Interview: “It gives you the real little tips, the hints, and what you missed, which is great.”
Finding	Performance feedback (U)

Illustration	Interview: "If I see I'm not performing as well as my colleagues they must be doing something more than I am, so I need to be more aggressive."
Finding	Systematic consistent care (U)
Illustration	Interview: "I think the follow-ups— that's really impacted. Because it's all spelled out when they should come back. I think before I wasn't really thinking of, oh, in 3 months rather than 6 months or 4 months."

**Study: Orchard et al, 2019**	
Finding	Patient satisfaction (C)
Illustration	Interview: "The patients really quite enjoyed it and I quite enjoyed playing with a little machine too. I quite enjoyed the technology side of it."
Finding	Resistance to technology (U)
Illustration	Interview: "I'm probably technically challenged sometimes…I don't want to push any wrong buttons."
Finding	Added time (C)
Illustration	Interview: "It was something to be done on quieter days, not horrendous clinical days… when we were running behind time, you tended not to get the screening done because it was on top of what the consultation was about."
Finding	Screening champion (U)
Illustration	Interview: "A couple of GPs really took it on, while a couple of them found it a burden. A GP champion spoke to them and said 'we really need to be doing this'."
Finding	Led to quality improvement (C)
Illustration	Interview: "When I saw the data…my first reaction was that's actually a really good study."
Finding	Increased motivation (C)
Illustration	Interview: "Feedback's always great and timely feedback is particularly important. It is lovely to have been able to receive timely feedback each month, and we have enjoyed the friendly competition within our team!"
Finding	Reliability is key (C)
Illustration	Interview: "It's just so unreliable…that unless you've got someone like me who really is willing to give it a go…[others] don't have the time."

**Study: O'Reilley et al, 2014**	
Finding	Satisfaction (N)

Illustration	Authors: The percentage of positive responses (mostly agree and completely agree) was higher than that of negative responses (strongly disagree and somewhat disagree) in 6 of the 9 categories

**Study: Patel et al, 2018**	
Finding	Team work (C)
Illustration	Interview: "It helps our nurse, so then we ah look, this person hasn't had such and such for a while wo when you're doing a chronic disease management say for cardiovascular or diabetes or anything like that, that all shows up as well. And so then we can then say to Dr. XXX (main GP) well look this hasn't been done either because he might not have seen that patient in that last six months….so it's like feedback from the nurse and also the doctor as well."
Finding	Allocated person (U)
Illustration	Interview: "A good single person allocated, keep monitoring, keep going, these tools will be very, very good. Yeah."
Finding	Laborious data collection (U)
Illustration	Interview: "it's a bit of a nuisance and apart from the fact that it is beneficial in terms of putting figures down and calculating it, it appears as if I'm working for somebody else with no compensation and no recompense and it's very annoying sometimes, especially when the thing clogs my computer now."
Finding	Following up session (U)
Illustration	Interview: "I think it's just about that training. So, and then following up, you know, so for example, the doctor has training, you know, following up a month later just do we need not a re-training but, you know, any questions. What we find is most of the doctors when they go to the initial training session they've, you know, they may not even have a login so they've never actually seen Health Tracker at all…. they really benefit from having maybe a following up session."
Finding	Organisational mission and history (N)
Illustration	Authors: One of the ACCHSs (case 5) had prioritised the use of CQI processes over 10 years, and this was evident in strategy documents, staffing allocations and prior use of various CQI tools.
Finding	Leadership (N)
Illustration	Authors: when the motivated leader's interests were aligned with those of his trusted practice manager, then engagement in the new practice was enhanced
Finding	Post-trial period (N)
Illustration	Authors: It was clear from this study that support provided by the research team played a central role in driving engagement, and it is not surprising that

	there was a plateauing of trial outcomes in many cases once support was reduced in the post-trial period.

**Study: Peiris et al, 2011**	
Finding	Immediately there (U)
Illustration	Interview: "So I think it can only be a bonus: the fact that it's immediately there"
Finding	Blanket recommendations (U)
Illustration	Interview: "She's [already] had her cholesterol done and then it says 'cholesterol evaluation is recommended'…They're telling us to suck eggs repeatedly, and I don't like it."
Finding	Burdensome prompts (U)
Illustration	Interview: "I mean, there are already a lot of pop-up windows…and adding another one isn't going to get anyone very excited…Imagine how much better the software would be if it tracks the way you use a consultation"
Finding	Created quarantined discussions (C)
Illustration	Interview: "I think it was quite a good thing because you would finish the consultation about whatever that was about and then you'd almost have a separate time set for looking at cardiovascular risk…So having that piece of paper [the tool output] there gave you that conversation: 'well now we've finished everything, let's look at this'"
Finding	Distorted priorities (C)
Illustration	Interview: "One of the dangers I would see with this is the encouragement of game playing…So an electronic decision support module that is only related to cardiovascular disease…could lead you to focus on getting cholesterol and things done and perhaps forget immunisations or pap smears or the housing forms because that's what the computer is flashing up at you."
Finding	Use of colour (U)
Illustration	Interview: "Yeah and even a coloured diagram is really helpful in being able to say, 'Look, the computer says it's true. This isn't just me making up words around diabetes. Look, this is going into orange – this says ''high'' in red'. And there's almost an emotional response to the colours that come back that is actually really useful compared to me saying, 'look people with diabetes have heart attacks and strokes'."
Finding	Perceived external authority (U)
Illustration	Interview: "They [patients] come for condition X and the machine is tapping me on the shoulder and saying, 'by the way, look at this'. Well I suppose one thing that crosses my mind is embarrassment. If it was so bad that you'd missed it and it said, 'Hey you need to prescribe this like now', I would be thinking 'who's running the show, the machine or me?' … Somewhere in my

	past I’ve always wanted to make sure that the doctor is the one who is making the decisions.
Finding	Conflicting guidelines (C)
Illustration	Interview: "It could get confusing, because I think you’d end up having the programmers or the decision-makers around the computer system becoming experts…and then there would be all sorts of quotes from professors or medical newspapers saying, ‘Well, that’s not the recommendation and we shouldn’t be doing that’. It leads to confusing messages because there isn’t actually the consensus around something until it hits the guidelines"
Finding	Value (N)
Illustration	Interview: "In general practice [tools are] not that important…I think most of us, we really treat on empirical grounds.We have a feeling that this person is at higher risk, and we just treat them…I don’t seem to have many slip through my fingers, because I see them a couple of times a year for coughs and colds etc"

**Study: Praveen et al, 2014**	
Finding	New knowledge (C)
Illustration	Interview: "Earlier, I just used to go and measure BP, but with this tablet, I came to know what was a normal reading and how the actual reading differs from normal readings"
Finding	Confidence (U)
Illustration	Interview: "First, we were afraid that there was the need to handle computers and touch screens, but later after training, we were able to understand it. After we did 1 or 2 tests, it became easy, and we can do it better now."
Finding	Communicating with patients (C)
Illustration	Interview: "It’s wonderful. I got better results than I expected...If patients see the risk bar, they understand very well that they have a high risk of CVD...We gained knowledge from this percentage display too...This is 100% beneficial to the doctor."
Finding	Downstream barriers (U)
Illustration	Interview: "If we refer somebody and if the doctor examines them, then the patients would respect us and it will be good...if it is not done, there may be a problem. If the doctor won’t examine or is not available when we send patients, then we will lose impression."
Finding	Skill expansion (U)
Illustration	Interview: "For people at my level to inform others about their health conditions is really a fortunate thing. Usually for these things, only specialists can talk about them. But with this tablet we are able to tell them what are the

	present levels and what would be future levels (of risk), and to what extent one needs to take care..."

**Study: Raghu et al, 2015**	
Finding	Usability (N)
Illustration	Survey: In over 72% of the screening procedures performed, the mobile application was found easy to use for that particular procedure.
Finding	Rating (N)
Illustration	Survey: No user gave the application a rating below 3 on a scale of 4, with 4 being most useful and 1 being least useful
Finding	Risk factor collection (N)
Illustration	Survey: In less than 2% of the procedures performed, the ASHAs recorded difficulties with collection of risk factors (such as blood pressure, height and weight, blood glucose).

**Study: Regan et al, 2017**	
Finding	Utilization (N)
Illustration	Survey: The majority of PCPs were interested in a CDS tool for CKD patients at the point of care and the majority of PCPs utilized the CDS at least sometimes during appointments
Finding	Knowledge (N)
Illustration	Authors: PCPs increased their overall knowledge of CKD stages, diagnostic criteria for CKD, and the significance of the ACR value in the detection of underlying renal pathologic abnormalities
Finding	Usefulness (N)
Illustration	Survey: 53% rated 'very much' for usefulness of a CKD CDS between matched pairs

**Study: Romero-Brufau et al, 2020**	
Finding	Patient preparedness (N)
Illustration	Survey: more users indicated that patients were better-prepared to manage diabetes following implementation of the AI-based CDS ($p=0.04$).
Finding	Care coordination (C)
Illustration	Survey: staff felt that care was better coordinated following implementation ($p<0.01$).
Finding	Familiarity (C)

Illustration	Survey: Following implementation of AI, users expressed significantly greater familiarity with AI ($p<0.01$)
Finding	Attitude towards intervention (N)
Illustration	Survey: There was no difference in attitudes about identifying the right interventions for the right patients
Finding	Attitude towards complications (N)
Illustration	Survey: There was no difference in attitudes about intervening to reduce complications of diabetes
Finding	Attitude toward complication reduction (N)
Illustration	Survey: There was no difference in attitudes about the clinic's effectiveness at reducing complications.
Finding	AI does not understand their jobs (C)
Illustration	Survey: users were more likely to feel that AI does not "understand" their jobs sufficiently ($p<0.01$).
Finding	Potential (N)
Illustration	Survey: Only one third (34 %) of users felt that the system had the potential to be useful with improvements.
Finding	Team dialogue (N)
Illustration	Survey: The most commonly-listed facilitator was that the CDS promoted team dialog about patient needs (52%).
Finding	Intervention personalisation (N)
Illustration	Survey: 24 % were dissatisfied that the interventions were too similar between different patients and therefore were not sufficiently tailored to each patient.

**Study: Shemeikka et al, 2015**	
Finding	Usefulness (C)
Illustration	Survey: overall usefulness of the CDSS was rated 4.4/6
Finding	Increases attention (N)
Illustration	Survey: increases attention on patients with impaired renal function (5.1/6)
Finding	Helps prescribe appropriate drugs (N)
Illustration	Survey: helps prescribe appropriate drugs (4.4/6)
Finding	References (N)
Illustration	Survey: perceived usefulness of references (PubMed-links etc) (2.9/6)

Finding	Dosing recommendations (N)
Illustration	Survey: perceived usefulness of dosing recommendation classification (4.7/6)

**Study: Singh et al, 2018**	
Finding	Follow-up care (N)
Illustration	Authors: Physicians in private hospitals had mixed feelings about how receptive people with diabetes would be to receiving reminders for follow-up care, some fearing that they would think their clinic was trying to make money from more frequent visits
Finding	Need (N)
Illustration	Authors: As diabetes specialists, there was little perceived need for the DSS because physicians felt that they were aware of treatment targets and guidelines for care.
Finding	Usage (N)
Illustration	Authors: At least one clinic reported not using the DSS prompts at all in their practice.
Finding	Safety-net (N)
Illustration	Authors: The remaining clinics viewed the treatment prompts as a safety-net system that would replace the junior colleagues who usually take histories and assist in consultations with people with diabetes
Finding	Physician role (N)
Illustration	Authors: Physicians recognized that the processes required by the DS-EHR system can be time consuming, but because the care coordinator's managed those tasks, they found their role did not change much with this intervention.
Finding	Missed tasks (N)
Illustration	Interview: "The biggest problem is that this hospital has such a big OPD [outpatient department] . . .[that], you would look at the patient ..and within 2 or 3 minutes you would come to a conclusion as to what needs to be done, in this there are times when you miss doing things which were supposed to be done in that particular time"
Finding	Space saving (C)
Illustration	Interview: "DS-EHR is useful, it keeps all the data, saves space"
Finding	Historical data (U)
Illustration	Interview: "If the patient comes to me today with the investigation, then I examine him today and based on today's report. . .the fact that I have historical data has helped me make better decisions"

Study: Sperl-Hillen et al, 2018	
Finding	Discussion initiation (C)
Illustration	Survey: 94% reported CDS system helped them initiate discussions about CV risk
Finding	Saved time (C)
Illustration	Survey: 93% reported saved time when talking about CV risk with patients
Finding	Supported shared decision making (C)
Illustration	Survey: 95% reported supported shared decision making
Finding	Influenced treatment (C)
Illustration	Survey: 89% reported CDS influenced treatment recommendations
Finding	Interface (C)
Illustration	Survey: 85% reported that their patients liked the CDS patient interface and associated activities

Study: Vedanthan et al, 2015	
Finding	Preference (N)
Illustration	Authors: 5/6 nurses preferred using the tablet to the paper form. DESIRE helped to organize their files and provided a helpful offsite backup.
Finding	Patient privacy (N)
Illustration	Authors: nurses frequently stated that using DESIRE enhanced patient privacy
Finding	Ease of use (C)
Illustration	Interview: "I thought it would really give a lot of hard time, going through the paper work then the tablet, but with time we've found it easier to use the tablet than the paper work."
Finding	Confidence (N)
Illustration	Authors: Several of the barriers to implementation detailed above, including network and server problems, decreased nurses' confidence in the device.
Finding	Faster than going to the files (C)
Illustration	Interview: "I think when I use the tablet am able to trace that patient faster than going to the files, looking for the files, feeding the files"
Finding	Relationship reduced (C)
Illustration	Interview: "the relationship now has reduced simply because we concentrate too much in the gadget. The patient likes to talk to you directly but you, you just concentrate in the gadget."

**Study: Wan et al, 2012**	
Finding	Good reminder (U)
Illustration	Interview: "It gives us a … very quick summary and … very good reminder of what needs to be done."
Finding	Visual aide (U)
Illustration	Interview: "illustrations help the patient to link the information to something else in their brain and they're more likely to remember it"
Finding	Did not see a need (U)
Illustration	Interview: "I think it doesn't affect someone if your knowledge of diabetes is substantial "
Finding	Costing time (U)
Illustration	Interview: "I think it takes a bit longer because it's you know a lot of ticking and putting things in"
Finding	Interfered with communication (U)
Illustration	Interview: "It makes me focus more on the computer."
Finding	Introductory training (C)
Illustration	Interview: "I think it does require a fairly good introduction, introductory session to show where the effectiveness of the tool"
Finding	Wrong alerts (C)
Illustration	Interview: "e.g. sometimes alerts everything in red (as abnormal), still no change even when changing the values manually"
Finding	Information not included (C)
Illustration	Interview: "e.g. information on vaccinations, cardiovascular risk"
